# Ibrutinib as monotherapy versus combination therapy in Chinese patients with relapsed/refractory mantle cell lymphoma: A multicenter study

**DOI:** 10.1002/cam4.4765

**Published:** 2022-04-19

**Authors:** Yuchen Zhang, Panpan Liu, Jun Cai, Hongmei Jing, Liqun Zou, Huiqiang Huang, Yuanbin Wu, Wenyu Li, Liye Zhong, Xueli Jin, Xu Ye, Ru Feng, Huilai Zhang, Liling Zhang, Lie Lin, Xiuhua Sun, Yuyang Tian, Zhongjun Xia, Zhiming Li, He Huang, Yi Xia, Qingqing Cai

**Affiliations:** ^1^ State Key Laboratory of Oncology in South China, Collaborative Innovation Center for Cancer Medicine Sun Yat‐sen University Cancer Center Guangzhou P.R. China; ^2^ Department of Medical Oncology Sun Yat‐sen University Cancer Center Guangzhou P.R. China; ^3^ Department of Hematology Peking University Third Hospital Beijing P.R. China; ^4^ Department of Oncology West China Hospital, Sichuan University Chengdu P.R. China; ^5^ Department of Hematology Guangdong Province Traditional Chinese Medical Hospital Guangzhou P.R. China; ^6^ Division of Lymphoma, Department of Clinical Oncology Guangdong Provincial People's Hospital, Guangdong Academy of Medical Sciences Guangzhou P.R. China; ^7^ Department of Hematology Guangdong Provincial People's Hospital, Guangdong Academy of Medical Sciences Guangzhou P.R. China; ^8^ Department of Hematology The Second Affiliated Hospital of Zhejiang University School of Medicine Hangzhou P.R. China; ^9^ Department of Hematology The Second Affiliated Hospital of Guangzhou Medical University Guangzhou P.R. China; ^10^ Department of Hematology Nanfang Hospital of Nanfang Medical University Guangzhou P.R. China; ^11^ Department of Lymphoma Tianjin Medical University Cancer Hospital and Institute Tianjin P.R. China; ^12^ Cancer Center, Union Hospital, Tongji Medical College Huazhong University of Science and Technology Wuhan P.R. China; ^13^ Department of Hematology Hainan General Hospital Haikou P.R. China; ^14^ Myeloma and Lymphoma Research Center Second Affiliated Hospital of Dalian Medical University Dalian P.R. China; ^15^ Department of Hematology Hainan Cancer Hospital Haikou P.R. China; ^16^ Department of Hematologic Oncology Sun Yat‐sen University Cancer Center Guangzhou P.R. China

**Keywords:** combination therapy, ibrutinib, mantle cell lymphoma, relapsed/refractory

## Abstract

**Background:**

Ibrutinib has revolutionized the treatment of mantle cell lymphoma (MCL). Both ibrutinib monotherapy and ibrutinib‐based combination therapy are important salvage options for patients with relapsed/refractory (R/R) MCL. The real‐world efficacy and safety profile of the two strategies in Chinese patients with R/R MCL remain unclarified.

**Methods:**

In the present study, data of 121 R/R MCL patients who received either ibrutinib monotherapy (*N =* 68) or ibrutinib combination therapy (*N =* 53) in 13 medical centers in China were retrospectively reviewed.

**Results:**

With a median follow‐up of 20.5 months, the overall response rate was 60.3% versus 84.9% (*p* = 0.003), complete remission rate was 16.2% versus 43.4% (*p* < 0.001), and median progression‐free survival (PFS) was 18.5 months (95% confidence interval [CI], 12.1–21.8) vs. 30.8 months (95% CI, 23.5‐NR) (hazard ratio, 0.53 [95% CI, 0.30–0.93]; *p* = 0.025), with ibrutinib monotherapy and ibrutinib‐based combination therapy, respectively. Subgroup analysis showed that patients with male gender, no refractory disease, Ki67 <30%, previous line of therapy = 1, non‐blastoid subtype, and the number of extranodal sites involved <2 might benefits more from the combination therapy. Treatment‐emergent adverse events were similar, except for a higher incidence of all grade neutropenia in the ibrutinib combination group (12.7% vs. 32.0%, *p* = 0.017).

**Conclusions:**

Ibrutinib combination therapy demonstrated potentially superior efficacy and comparable tolerability to ibrutinib monotherapy. Ibrutinib‐based combination therapy could be one of the prominent treatment options for R/R MCL patients.

## Graphical abstract

Compared to ibrutinib monotherapy, ibrutinib‐based combination therapy showed potentially superior efficacy for relapsed/refractory mantle cell lymphoma patients, with similar safety profile. Combination therapy based on ibrutinib could be considered as one of the prominent treatment options for this group of patients.

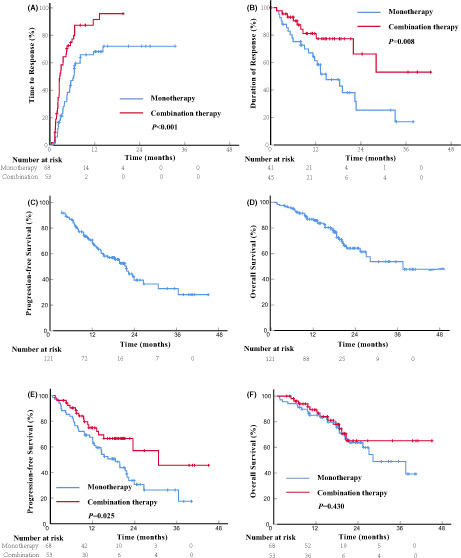

## 1 INTRODUCTION

Mantle cell lymphoma (MCL) is a distinct subtype of non‐Hodgkin lymphoma that is characterized by reciprocal translocation t(11;14)(q13; q32), resulting in Cyclin D1 overexpression in naïve pregerminal‐center B cells.^1^ Ibrutinib is an oral Bruton tyrosine kinase (BTK) inhibitor used widely to treat relapsed/refractory (R/R) MCL.^2^, ^3^ The efficacy and cytotoxicity of single‐agent ibrutinib have been evaluated in prospective phase II and III clinical trials.^4^, ^5^ A phase II clinical trial evaluating salvage therapy with ibrutinib in 111 patients with MCL reported an overall response rate (ORR) of 68% and complete remission (CR) rate of 21%.^4^ Follow‐ups at 27 months demonstrated that the efficacy remained durable with a favorable safety profile.^6^ In addition, a phase III trial presented superior efficacy and tolerability of ibrutinib to temsirolimus.^5^ Additionally, several retrospective studies from the United States, Europe, and South Korea have confirmed the effectiveness and safety of ibrutinib monotherapy in different patient populations in the “real‐world” setting.^7^, ^8^, ^9^, ^10^, ^11^, ^12^, ^13^, ^14^

Ibrutinib was approved by the National Medical Products Administration (NMPA‐China) to treat patients with MCL who had received at least one prior therapy as of August 2017. To the best of our knowledge, data on the clinical outcomes and safety profile of ibrutinib in Chinese MCL patients is lacking. This may be attributed to the rarity of the disease and the relatively short period following approval of ibrutinib in China.

Although ibrutinib has transformed the treatment of R/R MCL, there are still several limitations when used as a single agent, including a low CR rate, short PFS, and development of resistance due to mutations of BTK or other molecular targets in the B‐cell receptor pathway.^15^ Therefore, there has been a growing interest in combining ibrutinib with other agents. Several phase II studies evaluating ibrutinib combination with rituximab, lenalidomide, or venetoclax in patients with R/R MCL reported CR rates of approximately 40%–70%.^16^, ^17^, ^18^, ^19^ According to the cross‐trial comparisons, ibrutinib‐based combination therapy seemed to show a superior CR rate compared to ibrutinib monotherapy. Currently, there is limited data on the efficacy, toxicity, and the specific patient population expected to benefit from ibrutinib‐based combination therapies in clinical practice. Therefore, further studies evaluating ibrutinib‐based combination therapies are needed.

Due to the lack of direct comparative data from randomized clinical trials, we conducted a nationwide, multicenter, retrospective analysis comparing the efficacy and toxicity of ibrutinib monotherapy and ibrutinib‐based combination therapy in R/R MCL patients in China.

## 2 METHODS

### 2.1 Patients and study design

Adult R/R MCL patients initiated on ibrutinib monotherapy or ibrutinib‐based combination therapy between August 2017 and December 2020 were included in this study. Inclusion criteria were as follows: (1) pathologically confirmed MCL according to the 2016 World Health Organization classification of lymphoma^20^; (2) age ≥18 years; (3) relapsed or refractory to the most recent line of treatment; 4) received ibrutinib monotherapy or ibrutinib combination therapy. The exclusion criteria included: (1) previously treated with BTK inhibitors; (2) participated in MCL‐related or ibrutinib‐related clinical trials; (3) missing response assessment after treatment. Patients were divided into the ibrutinib monotherapy group or ibrutinib combination therapy group according to the received medications. Patients in monotherapy group received ibrutinib until disease progression, unacceptable toxicity, or physician/patients' choice. Patients in combination group received ibrutinib‐based combination therapy for 4–8 cycles. Patients who achieved at least stable disease (SD) could receive maintenance therapy, while who experienced disease progression during treatment would receive next‐line treatment. Decisions regarding combination therapy regimens, hematopoietic stem cell transplantation, and use of maintenance therapy after inductive ibrutinib‐based combination therapy were primarily made by the treating physicians, taking individual patients' willingness into account.

Thirteen medical centers in China participated in this study, including (1) Sun Yat‐sen University Cancer Center; (2) Peking University Third Hospital; (3) West China Hospital, Sichuan University; (4) Guangdong Provincial People's Hospital; (5) Guangdong Province Traditional Chinese Medical Hospital; (6) Tianjin Medical University Cancer Hospital and Institute; (7) Cancer Center, Union Hospital, Tongji Medical College; (8) The Second Affiliated Hospital of Zhejiang University School of Medicine; (9) The Second Affiliated Hospital of Guangzhou Medical University; (10) Nanfang Hospital of Nanfang Medical University; (11) Second Affiliated Hospital of Dalian Medical University; (12) Hainan General Hospital; and (13) Hainan Cancer Hospital.

The study protocol was approved by the institutional review board, and the study was performed in compliance with the Declaration of Helsinki. The included patients provided written informed consent.

### 2.2 Outcomes

Data on ORR, CR rate, time to response (TTR), DOR, PFS, overall survival (OS), reasons for ibrutinib discontinuation, and incidence of treatment‐emergent adverse events (TEAEs) were collected. Response to ibrutinib therapy was assessed according to the 2014 Lugano criteria based on medical records in each medical center.^21^ TTR was defined as the time from the start of ibrutinib therapy to attaining the first objective response. DOR was defined as the time from the first objective response to disease progression or death due to any cause. PFS was defined as the time from initiation of ibrutinib therapy to disease progression or death from any cause. OS was defined as the time from the start of ibrutinib therapy to death from any cause. For patients without reported events, the time‐to‐event was censored on the date of the last follow‐up. The refractory disease was defined as failure to achieve CR to the most recent regimen before receiving ibrutinib. Hematological AEs were evaluated according to the National Cancer Institute Common Terminology Criteria for Adverse Events (NCI CTC AE version 5.0). Non‐hematological AEs were not graded due to a lack of relevant data.

### 2.3 Targeted NGSand sequencing data analysis

Samples were obtained from formalin‐fixed paraffin‐embedded (FFPE) tissue sections after relapse of the last line of therapy before ibrutinib initiation. Tumor samples with >10% tumor cells identified by immunohistochemistry were eligible for next‐generation sequencing (NGS) analysis. Genomic DNA from FFPE samples was extracted using the QIAamp DNA FFPE Tissue kit (Qiagen). Sequencing libraries were prepared using the KAPA Hyper Prep Kit (KAPA Biosystems). Hybridization enrichment was performed using customized xGen lockdown probes (Integrated DNA Technologies) targeting 446 leukemia‐ and lymphoma‐related genes. Dynabeads M‐270 (Life Technologies) and xGen Lockdown hybridization and wash Kit (Integrated DNA Technologies) were used for capture reaction. Captured libraries were amplified by polymerase chain reaction (PCR). The mean depth of coverage sequencing was 1000×.

Trimmomatic was used for quality control of FASTQ file. The leading and trailing low quality (quality reading <20) or *N* bases were removed. Then, the paired‐end reads were aligned to the human reference genome (hg19) using Burrows‐Wheeler Aligner. Picard was used to remove PCR duplicates. Genome Analysis Toolkit was used for local realignment around indels and recalibration of base quality score. Matched normal control of the germline DNA was not available due to the retrospective nature of this study. Somatic mutations were first called from each sample (the filtering criteria were a variant frequency of ≥0.5% and ≥5 supporting reads from both directions). Common single‐nucleotide polymorphisms (SNPs) were filtered out if the population frequency was >1% in the 1000 Genomes Project or the Exome Aggregation Consortium 65,000‐ exome database. The mutation list was further filtered by an in‐house list of recurrent artifacts and common SNPs based on blood samples sequenced with the same gene panel in approximately 500 Chinese patients with malignancies. Single‐nucleotide variations/indels were annotated with ANNOVAR, and were manually checked on the Integrative Genomics Viewer. Copy number variations were detected using in‐house‐developed software.

### 2.4 Statistical analysis

Baseline characteristics were analyzed by descriptive statistics. Categorical variables were presented as the number and percentage of individuals in each category. Chi‐square test, Fisher exact test, or Wilcoxon rank‐sum tests were used to compare proportions between two treatment groups. All time‐to‐event endpoints, including PFS, OS, TTR, and DOR, were estimated using the Kaplan–Meier survival method and compared by a Log‐rank test. The hazard ratio (HR) for the combination therapy group relative to the monotherapy group and the 95% confidence interval (CI) were calculated using the Cox regression model. Statistical analyses were performed using SAS 9.4. A *p*‐value of <0.05 was set as statistically significant.

## 3 RESULTS

### 3.1 Baseline characteristics and treatment

A total of 121 R/R MCL patients met the eligibility criteria. There were 68 and 53 patients in ibrutinib monotherapy group and ibrutinib‐based combination therapy group, respectively. A flow diagram of the case selection process is presented in Figure S1. Table 1 depicts the baseline characteristics of the enrolled patients at the time of ibrutinib initiation. Over 60% of patients received ibrutinib following failure of the first‐line treatment. The first‐line and the most recent front‐line therapies before ibrutinib initiation are summarized in Table S1. The combination therapy group had a smaller proportion of patients with age ≥60 (*p* = 0.033) than the monotherapy group. No statistically significant differences were observed in other baseline characteristics between the two groups.

**TABLE 1:** Baseline characteristics at ibrutinib initiation

	Entire cohort *n* = 121	Monotherapy *n* = 68	Combination therapy *n* = 53	*p* value^a^
Age				
Median (range), years	60 (34–81)	63 (34–81)	56 (42–80)	
≥60 years	68 (56.2%)	44 (64.7%)	24 (45.3%)	0.033
<60 years	53 (43.8%)	24 (35.3%)	29 (54.7%)	
Gender				
Male	85 (70.2%)	45 (66.2%)	40 (75.5%)	0.267
Female	36 (29.8%)	23 (33.8%)	13 (24.5%)	
ECOG‐PS				
0–1	91 (75.2%)	53 (77.9%)	38 (71.7%)	0.623
≥2	30 (24.8%)	15 (22.1%)	15 (28.3%)	
Ann Arbor stage				
I–II	15 (12.4%)	7 (10.3%)	8 (15.1%)	0.427
III–IV	106 (87.6%)	61 (89.7%)	45 (84.9%)	
Refractory disease^b^				
Yes	57 (47.1%)	32 (47.1%)	25 (47.2%)	0.990
No	64 (52.9%)	36 (52.9%)	28 (52.8%)	
Number of previous therapy lines				
1	76 (62.8%)	41 (60.3%)	35 (66%)	0.197
2	22 (18.2%)	16 (23.5%)	6 (11.3%)	
≥3	23 (19%)	11 (16.2%)	12 (22.6%)	
Previous ASCT				
Yes	8 (6.6%)	3 (4.4%)	5 (9.4%)	0.296
No	113 (93.4%)	65 (95.6%)	48 (90.6%)	
Histology subtype^c^				
Blastoid	17 (15.0%)	7 (11.5%)	10 (19.2%)	0.250
Non‐blastoid	96 (85.0%)	54 (88.5%)	42 (80.2%)	
Ki67^c^				
<30%	55 (47.8%)	32 (50.8%)	23 (44.2%)	0.483
≥30%	60 (52.2%)	31 (49.2%)	29 (55.8%)	
Simplified MIPI				
Low risk	52 (43.0%)	26 (38.2%)	26 (49.1%)	0.381
Intermediate risk	49 (40.5%)	31 (45.6%)	18 (34.0%)	
High risk	20 (16.5%)	11 (16.2%)	9 (17.0%)	
MIPI‐c^d^				
Low risk	19 (16.5%)	10 (15.9%)	9 (17.3%)	0.628
Low‐intermediate risk	52 (45.2%)	27 (42.9%)	25 (48.1%)	
Intermediate‐high risk	37 (32.2%)	23 (36.5%)	14 (26.9%)	
High risk	7 (6.1%)	3 (4.8%)	4 (7.7%)	
Bulky mass ≥5 cm				
Yes	43 (35.5%)	23 (33.8%)	20 (37.7%)	0.656
No	78 (64.5%)	45 (66.2%)	33 (62.3%)	
Number of extranodal involvement				
0–1	77 (63.6%)	46 (67.6%)	31 (58.5%)	0.299
≥2	44 (36.4%)	22 (32.4%)	22 (41.5%)	
CNS involvement				
Yes	4 (3.3%)	1 (1.5%)	3 (5.7%)	0.318
No	117 (96.7%)	67 (98.5%)	50 (94.3%)	

*Note*: Data are shown as number (%) or median (range). The sum of some percentages may not equal 100% because of rounding.

Abbreviations: ASCT, autologous stem cell transplantation; CNS, central nervous system; ECOG‐PS, Eastern Cooperative Oncology Group performance status; MIPI, mantle‐cell lymphoma international prognostic index; MIPI‐c, combined mantle‐cell lymphoma international prognostic index.

^a^ The *p* value of comparison between ibrutinib monotherapy group and ibrutinib‐containing combination therapy group.

^b^ Refractory disease was defined as failure to achieve CR to the most recent regimen before receiving ibrutinib.

^c^ Eight patients were lack of histology subtype data and six patients were lack of Ki67 data.

^d^ Six patients were excluded from the analysis of MIPI‐c due to lack of Ki67 data.

### 3.2 Treatment and response

Forty‐five patients (84.9%) of the 53 patients in the combination therapy group received a chemotherapy‐free regimen. Ibrutinib + rituximab (IR) (32/53, 60.4%) was the most common combined therapy, followed by ibrutinib + rituximab + lenalidomide (IR^2^) (10/53, 18.9%). A summary of the regimens in the combination therapy group is presented in Table S2. During the combination therapy, eight patients experienced disease progression requiring a change in treatment. For the remaining 45 patients who achieved at least SD and did not develop disease progression after 4–8 cycles of combination therapy, 91.1% (41/45) received maintenance therapy afterward. The maintenance regimens included ibrutinib monotherapy (*N =* 29), IR (*N =* 10), rituximab (*N =* 1), and rituximab + lenalidomide (*N =* 1).

**FIGURE 1:**
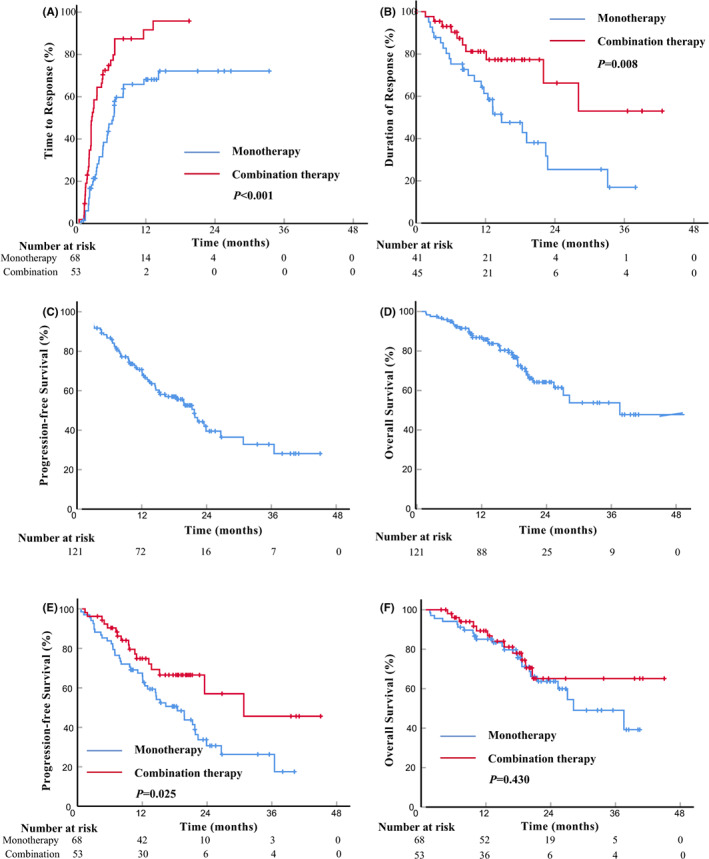
Kaplan–Meier curves. (A) Time to response. (B) Duration of response. (C) Progression‐free survival. (D) Overall survival. (E) Progression‐free survival of monotherapy group and combination therapy group. (F) Overall survival of monotherapy group and combination therapy group. Log‐rank *p* values are shown

The treatment response of the entire cohort and the two treatment groups is summarized in Table 2. The best ORR of the entire cohort was 71.1%, with a CR rate of 28.1% and a partial remission rate of 43.0%. The combination therapy group showed significantly higher ORR and CR rates than the monotherapy group (ORR, 84.9% vs. 60.3%, *p* = 0.003; CR rate, 43.4% vs. 16.2%, *p* < 0.001). Substantially fewer patients in the combination therapy group achieved SD (9.4% vs. 30.9%, *p* = 0.004). In patients who received chemotherapy‐free combination regimens, the ORR and CR rates were 84.4% and 44.4%, respectively. The median TTR for the monotherapy and combination groups were 6.3 months (95% CI, 4.6–8.1 months) and 2.6 months (95% CI, 2.4–3.5 months), respectively (Figure 1A). For the 86 patients who responded to treatment, the median DOR for the monotherapy group was 14.8 months (95% CI, 11.2–22.7 months), and not reached in the combination group (Figure 1B). The differences in TTR and DOR between the two treatment groups were statistically significant (TTR, *p* < 0.001; DOR, *p* = 0.008).

**TABLE 2:** Response to ibrutinib monotherapy and ibrutinib‐containing combination therapy

Response	Entire cohort (*n* = 121)	Monotherapy (*n* = 68)	Combination therapy (*n* = 53)	*p* value^a^
ORR (CR + PR)	86 (71.1%)	41 (60.3%)	45 (84.9%)	0.003
CR	34 (28.1%)	11 (16.2%)	23 (43.4%)	<0.001
PR	52 (43.0%)	30 (44.1%)	22 (41.5%)	0.774
SD	26 (21.5%)	21 (30.9%)	5 (9.4%)	0.004
PD	9 (7.4%)	6 (8.8%)	3 (5.7%)	0.730

*Note*: Data are shown as number (%).

Abbreviations: CR, complete remission; ORR, objective response rate; PD, progressive disease; PR, partial remission; SD, stable disease.

^a^ The *p* value of comparison between ibrutinib monotherapy group and ibrutinib‐containing combination therapy group.

### 3.3 Survival outcomes

With a median follow‐up of 20.5 months (95% CI, 18.3–22.6 months), the estimated median PFS of the entire cohort was 21.7 months (95% CI, 15.2–26.6 months), with 1‐ and 2‐year PFS of 70.7% and 39.5%, respectively (Figure 1C). The median OS was 37.6 months (25.4 months‐not estimable [NE]), with 1‐ and 2‐year OS of 86.9% and 64.2%, respectively (Figure 1D). The combination therapy group had a superior PFS compared to the monotherapy group (median PFS, 30.8 months [95% CI, 23.5 months‐NE] vs. 18.5 months [12.1–21.8 months]; HR, 0.53 [95% CI, 0.30–0.93]; *p* = 0.025) (Figure 1E). However, no OS benefit was observed with the combination therapy group (median OS, not reached [95% CI, 20.6 months‐NE] vs. 28.2 months [21.5 months‐NE]; HR, 0.77 [95% CI, 0.39–1.55]; *p* = 0.430) (Figure 1F). Fifty‐nine patients (42 in monotherapy group, and 17 in combination therapy group) had experienced treatment failure. The detailed information on subsequent therapies was recorded in 50 patients (Table S3).

**FIGURE 2:**
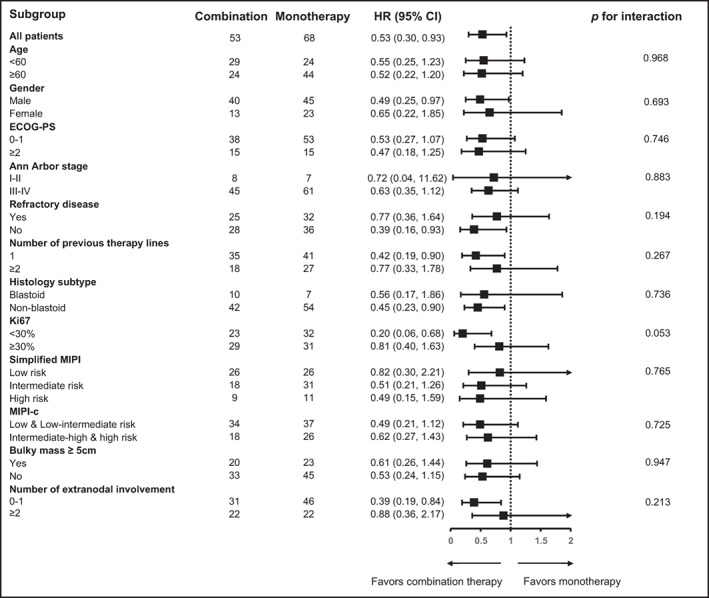
The forest plot of subgroup analysis for progression‐free survival. CI, confidence interval; ECOG‐PS, Eastern Cooperative Oncology Group performance status; HR, hazard ratio; MIPI, mantle‐cell lymphoma international prognostic index; MIPI‐c, combined mantle‐cell lymphoma international prognostic index

We also evaluated the PFS benefit of combination therapy versus monotherapy in different subgroups according to baseline characteristics (Figure 2). Several subgroups were combined or eliminated because of the small sample size. Statistically, significant PFS benefits were observed in patients with male gender, no refractory disease, Ki67 <30%, previous line of therapy = 1, non‐blastoid subtype, and the number of extranodal sites involved <2.

### 3.4 Ibrutinib discontinuation and adverse events

At the last follow‐up, 67 patients had discontinued ibrutinib‐based treatment, principally because of disease progression (50/67, 74.6%). Other reasons for ibrutinib discontinuation included: AEs (9/67, 13.4%; pulmonary infection, *N =* 2; bleeding, *N =* 3; second malignancies, *N =* 3; ventricular premature contractions and palpitations, *N =* 1), physician or patient choice (4/67, 6.0%), death unrelated to MCL (1/67, 1.5%), and unknown reason (3/67, 4.5%).

Complete information on TEAEs was recorded in 105 patients from six participating medical centers (Table 3). A significantly higher incidence of all grade neutropenia was observed in the combination group (32.0% vs. 12.7%, *p* = 0.017) compared with the monotherapy group. There were no other statistically significant differences in all grade or grade 3–4 TEAEs between the two groups. Of the 15 patients with an electrocardiogram (ECG) abnormality, atrial fibrillation was detected in only two of the patients. Other abnormalities included atrial (*N =* 11) and ventricular premature contractions (*N =* 2). No significant difference in incidence of ECG abnormality with ibrutinib dose was observed (*p* = 0.413) (Table S4). Major bleeding events occurred in four patients: central nervous system hemorrhage (*N =* 2), grade 3 gastrointestinal hemorrhage (*N =* 1), and grade 3 subcutaneous hemorrhage (*N =* 1). There were no new safety signals in this study during ibrutinib treatment.

**TABLE 3:** Treatment emergent adverse events

	All grade			Grade 3–4		
	Monotherapy (*N* = 55)	Combination therapy (*N* = 50)	*p* ^a^	Monotherapy (*N* = 55)	Combination therapy (*N* = 50)	*p* ^a^
Hematological adverse events						
Neutropenia	7 (12.7%)	16 (32.0%)	0.017	1 (1.8%)	2 (4.0%)	0.604
Thrombocytopenia	19 (34.6%)	14 (28.0%)	0.471	5 (9.1%)	3 (6.0%)	0.718
Anemia	11 (20.0%)	9 (18.0%)	0.794	2 (3.6%)	5 (10.0%)	0.254
Non‐hematological adverse events						
Nausea/vomiting	10 (18.2%)	14 (28.0%)	0.232	–	–	–
Rash	7 (12.7%)	11 (22.0%)	0.208	–	–	–
Infection	13 (23.6%)	13 (26.0%)	0.779	–	–	–
Bleeding	9 (16.4%)	6 (12.0%)	0.523	–	–	–
ECG abnormality^b^	7 (12.7%)	8 (16.0%)	0.632	–	–	–

Data are shown as number (%). Non‐hematological AEs were not graded due to lack of relevant data.

Abbreviation: ECG, electrocardiogram.

^a^ The *p* value of comparison between ibrutinib monotherapy group and ibrutinib‐containing combination therapy group.

^b^ The ECG abnormality includes atrial premature contractions (*N* = 11), ventricular premature contractions (*N* = 2), and atrial fibrillation (*N* = 2).

Newly diagnosed secondary malignancies occurred in four patients (two patients in each group) after ibrutinib treatment: acute myeloid leukemia, myelodysplastic syndromes, nasopharyngeal carcinoma, and colorectal carcinoma. All four patients had received cyclophosphamide, anthracycline, vincristine, and prednisone‐based chemotherapy before ibrutinib treatment. The patients who were diagnosed with nasopharyngeal carcinoma had received autologous stem cell transplantation previously.

### 3.5 Mutation profiling by targeted NGS

In the exploratory analysis, 446 leukemia and lymphoma‐relevant genes detected by NGS were analyzed in 21 patients with available tumor samples. The heatmap of gene mutation profiles of the 21 patients is shown in Figure S2. Missense mutation was the major type of gene mutation. The four genes with the highest mutation frequency were *ATM* (13/21, 61.9%), *KMT2D* (8/21, 38.1%), *NSD2* (6/21, 28.6%), and *SMARCA4* (5/21, 23.8%).

## 4 DISCUSSION

In this retrospective study, we analyzed the treatment patterns, efficacy, and toxicity of ibrutinib treatment in Chinese patients with R/R MCL in a real‐world setting. Ibrutinib‐based combination therapy achieved a significantly superior response rate, TTR, DOR, and PFS, and demonstrated an acceptable safety profile compared with ibrutinib monotherapy. The subgroup analysis identified several subgroups of patients that could benefit from the combination therapy.

Table S5 has summarized the cross‐trial comparison of our study with previous observational studies. The response rates and survival outcomes of ibrutinib treatment in our study were largely comparable to previously published reports.^7^, ^8^, ^9^, ^10^, ^11^, ^12^, ^13^, ^14^ However, it has to be mentioned that over 60% of patients had only one line of prior therapy in our study population. When comparting studies including patients at first relapse or with most patients who had one line of prior therapy, it seems that the efficacy results in our study were relatively inferior.^9^, ^11^, ^13^ What's more, the efficacy data in different studies showed disparities. Several reasons might have contributed to the difference. First, each study focused on patients from a specific country, and it was unknown if the clinical efficacy of ibrutinib differs across various ethnicities, which needs to be further explored. Also, quite a lot of factors that might influence efficacy varies in different countries and medical centers, such as healthcare condition, economic level, supportive care, surveillance of toxicity, patients' medication compliance, etc. Taken together, ibrutinib is an effective drug for R/R MCL after worldwide validation in real‐world setting.

The synergistic anti‐tumor effect of ibrutinib with other agents has been demonstrated in several pre‐clinical studies.^22^, ^23^, ^24^, ^25^ Prior clinical trials have reported promising efficacy with combination therapy.^16^, ^17^, ^18^, ^19^ In this study, the combination therapy group demonstrated significantly better response rates, survival outcomes, and time to obtain remission than ibrutinib monotherapy. Rapid response to combination therapy can quickly alleviate lymphoma‐related symptoms as well as reduce the tumor burden.

We conducted a subgroup analysis for PFS according to the baseline characteristics to determine if combination therapy could only benefit a specific group of Chinese MCL patients. Patients with heavily pretreated, more refractory, and highly aggressive diseases failed to show statistically significant PFS benefits with combination therapy. Most of the characteristics identified in our study (refractory disease, Ki67 ≥30%, previous line of therapy >1, blastoid subtype, and the number of extranodal sites involved ≥2) were previously reported as inferior prognostic factors for survival in patients who received ibrutinib treatment.^4^, ^5^, ^7^, ^8^, ^9^, ^10^, ^11^, ^17^ Nevertheless, it should be noted that all the included cases in our study were MCL patients from China, so the results might only be applicable to Chinese patients. There is a huge variation of disease characteristics in patients from different regions and ethnicities, which can affect the results of similar analysis. A curious finding from the subgroup analysis showed that male patients seemed to potentially benefit more from the combination therapy. To the best of our knowledge, no previous studies have demonstrated a gender‐dependent efficacy with ibrutinib treatment. However, some studies have reported gender discrepancies in survival outcomes of patients receiving immune‐checkpoint inhibitors, implying that gender might affect efficacy under certain circumstances.^26^, ^27^ This finding needs to be further elucidated.

Ibrutinib‐based combination therapy had a significantly higher rate of all‐grade neutropenia than ibrutinib monotherapy (*p* = 0.017). However, the neutropenia was manageable, and discontinuation due to neutropenia was not observed. We found no statistically significant difference in other toxicities between the two groups. Most patients reportehan ibrutinib monotherapy (*p* = 0.017). However, the neutropenia was manageable, and discontinuation due to neutropenia was not observed. We found no statistically significant difference in other toxicities between the two groups. Most patients reported mild to moderate adverse events, and severe (grade 3/4) toxicities were uncommon. Notably, the observed incidence rate of atrial fibrillation in the present study was lower than in previous clinical trials.^4^, ^5^, ^16^, ^17^, ^18^ This might be largely due to fact that ECG monitoring was not routinely performed in many of the included patients. In this study, no new safety signals were observed across the two treatment approaches. The benefits of promising efficacy and acceptable safety profile could support the broad use of ibrutinib for R/R MCL in the future.

Baseline characteristics of the two treatment groups were broadly similar except for a relatively younger age (<60 years) in ibrutinib combination group. Ibrutinib monotherapy group showed similar age distribution when compared to previous investigations, mostly with a median age of approximately 65–70 years.^4^, ^5^, ^7^, ^8^, ^9^, ^10^, ^11^, ^12^, ^16^, ^17^, ^18^ However, in our study, the median age of ibrutinib combination group was apparently lower, only with a median age of 56 years. It might suggest that clinicians had a greater tendency to give more intense regimens to younger MCL patients in routine clinical practice in China. Age has been identified as one of the prognostic factors in the mantle‐cell lymphoma international prognostic index.^28^ However, the value of age as an independent prognostic factor in the context of R/R MCL patients has not been fully verified. Also, to our knowledge, it has not been reported that age had an impact on response to ibrutinib treatment. Furthermore, considering that the median PFS was shorter than 3 years in both groups, the difference in age might not strongly influence the analysis of survival outcomes. For toxicity, although no significant differences were observed between different age groups (data not shown), randomized prospective clinical studies are warranted to further compare the two approaches. In general, the discrepancy of age in baseline characteristics might not influence the findings in this study.

This study further explored the correlation between gene alterations and clinical outcomes in MCL patients treated with ibrutinib‐containing therapy. The results showed similarities and differences in the mutation profiles of this study compared with previous reports.^29^, ^30^, ^31^ A recent study including a large Chinese MCL patient cohort showed a comparable mutation spectrum (a high mutation frequency of *ATM*, *TP53*, *NSD2*, *and KMT2D*) to this study.^32^ Different ethnic groups may also be associated with different mutation profiles in MCL.

The present study has several limitations. First, the retrospective nature of the study might introduce selection bias. Second, the subsequent treatment of patients who failed ibrutinib was not further discussed in this study due to missing treatment details. Third, despite the inclusion of patients from multiple centers, the sample size was still relatively small. For this reason, the results of subgroup analysis and NGS analysis should be interpreted with caution. Last, the maintenance therapy for the combination group was not uniform, which might have affected the results.

In conclusion, this multicenter retrospective study illustrated that the efficacy of ibrutinib‐based combination therapy was potentially superior to ibrutinib monotherapy, with comparable toxicity. Ibrutinib‐based combination therapy could be considered as one of the preferred treatment options for R/R MCL patients.

## CONFLICT OF INTEREST

The authors have declared no conflicts of interest.

## AUTHOR CONTRIBUTIONS

Qingqing Cai, Yuchen Zhang, and Panpan Liu were involved in conception and design of the study. Yuchen Zhang, Panpan Liu, and Jun Cai carried out methodology, data analysis, and interpretation. Qingqing Cai was involved in study supervision. All authors carried out acquisition of data, writing, review, and/or revision of the manuscript. They also reviewed the manuscript and approved submitting it.

## ETHICAL APPROVAL STATEMENT

The study was performed in compliance with the Declaration of Helsinki. The study protocol was approved by the institutional review board (B2020‐012‐01).

## Supporting information


Appendix S1

## Data Availability

The datasets used and/or analyzed during the current study are available from the corresponding author on reasonable request.
