# Supplementary material for: Ibrutinib as monotherapy versus combination therapy in Chinese patients with relapsed/refractory mantle cell lymphoma: A multicenter study
Source: Cancer Med. 2022 Apr 19;11(22):4134–45. doi: 10.1002/cam4.4765 (PMC9678091; doi:10.1002/cam4.4765)
Supplement: Supplementary file 1 — Appendix S1 [file CAM4-11-4134-s001.pdf]

Table S1. The previous first-line therapy regimen and the most recent front-line therapy before ibrutinib initiation.

Regimen	Number (%) N=121
**First-line therapy regimen**	
R-CHOP	61 (50.4%)
Alternating R-CHOP/R-DHAP	27 (22.3%)
CHOP	11 (9.1%)
R-HyperCVAD	6 (5.0%)
Rituximab + lenalidomide	3 (2.5%)
others	13 (10.7%)
**Most recent front-line therapy regimen**	
R-CHOP	38 (31.4%)
Alternating R-CHOP/R-DHAP	20 (16.5%)
Rituximab + lenalidomide	8 (6.6%)
CHOP	4 (3.3%)
VR-CAP	4 (3.3%)
others	47 (38.8%)

Data are shown as number (%). The sum of some percentages may not equal 100% because of rounding. R-CHOP, rituximab + cyclophosphamide, vincristine, doxorubicin, prednisone; R-CHOP/R-DHAP, rituximab, cyclophosphamide, doxorubicin, vincristine, prednisone/rituximab, dexamethasone, cytarabine, platinum; CHOP, cyclophosphamide, vincristine, doxorubicin, prednisone; R-HyperCVAD, cyclophosphamide, vincristine, doxorubicin, and dexamethasone alternating with high-dose methotrexate and cytarabine) + rituximab; VR-CAP, bortezomib, rituximab, cyclophosphamide, doxorubicin, and prednisone.

Table S2. The ibrutinib-containing regimens in combination therapy group.

Regimen	Number (%) N=53
**Chemotherapy-free regimen**	**45 (84.9%)**
Ibrutinib + rituximab	32 (60.4%)
Ibrutinib + rituximab + lenalidomide	10 (18.9%)
Ibrutinib + lenalidomide	2 (3.8%)
Ibrutinib + rituximab + bortezomib	1 (1.9%)
**Ibrutinib-containing chemotherapy regimen**	**8 (15.1%)**
Ibrutinib + bendamustine	2 (3.8%)
Ibrutinib + bendamustine + rituximab	2 (3.8%)
Ibrutinib + rituximab + chemotherapy	4 (7.6%)

Data are shown as number (%). The sum of some percentages may not equal 100% because of rounding.

Table S3. Subsequent therapy after ibrutinib failure.

Regimen	Monotherapy group (*N*=42)	Combination therapy group (*N*=17)
Bendamustine + rituximab	6 (14.3%)	3 (17.6%)
Chemotherapy	11 (26.2%)	7 (41.2%)
BTKi-based regimens	6 (14.3%)	2 (11.8%)
Lenalidomide ± rituximab	2 (4.8%)	1 (5.9%)
Others	3 (9.5%)	1 (5.9%)
Unknown	13 (31.0%)	3 (17.6%)

Data are shown as number (%). The sum of some percentages may not equal 100% because of rounding. BTKi, Bruton tyrosine kinase inhibitor.

Table S4. Relationship of ibrutinib dose and electrocardiogram abnormality.

**Ibrutinib dose**	**Electrocardiogram abnormality**		**Total**
	Yes	No	
560mg/d	10 (12.2%)	72 (87.8%)	82
< 560mg/d	5 (21.7%)	18 (78.3%)	23
Total	15	90	105

Data are shown as number (%).

Table S5. Cross-trial comparison of observational studies in R/R MCL patients treated with ibrutinib monotherapy.

References	Study design	Sample size	Median age (range), years	Lines of previous therapy (median [range])	ORR (%)	CR (%)	Median PFS (months)	Median OS (months)
**Current study**	Retrospective	68	63 (34-81)	1 (1-7)	60.3	16.2	18.5	28.2
**Broccoli et al.**[7]	Retrospective	77	65 (35-81)	3 (1-10)	36.4	18.2	12.9	16
**Epperla et al.**[8]	Retrospective	97	63 (39-87)	2 (1-8)	65	33	15	22
**McCulloch et al.**[9]	Retrospective	211	73 (33–96)	1	69	27	17.8	23.9
**Tucker et al.**[10]	Retrospective	65	67 (48–90)	2 (1–6)	NA	NA	12	18.5
**Yi et al.**[11]	Retrospective	88	71 (42-92)	1 (1-6)	64.8	NA	30.8	Not reached
**Jeon et al.**[12]	Retrospective	33	65 (40-79)	2 (1–4)	64	15	27.4	35.1
**Janssens et al.**[13]	Retrospective	71	74 (47-88)	1 (50.7%)<br>2 (31.0%)<br>⩾3 (18.3%)	93	32	22.3	39.4
**Slama et al.**[14]	Retrospective and prospective	106	74 (49-88)	1 (38.7%)<br>2 (31.1%)<br>⩾3 (30.2%)	76.4	NA	20.0	29.8

R/R, relapsed and refractory; MCL, mantle cell lymphoma; ORR, objective response rate; CR, complete remission; PFS, progression-free survival; OS, overall survival; NA, not available.

Figure S1

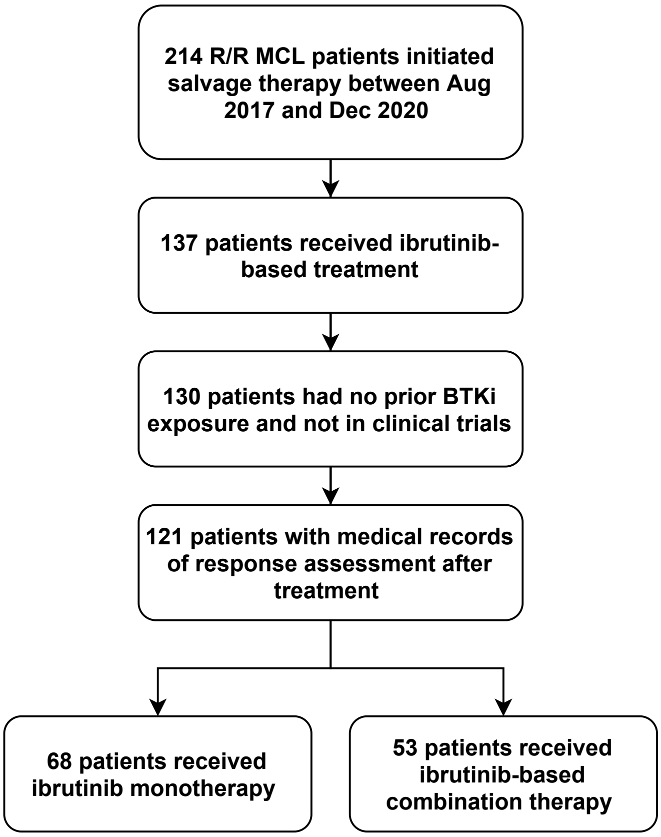

Figure S1. Flow diagram of the case selection process. R/R, relapsed/refractory; MCL, mantle cell lymphoma; BTKi, Bruton tyrosine kinase inhibitor.

Figure S2

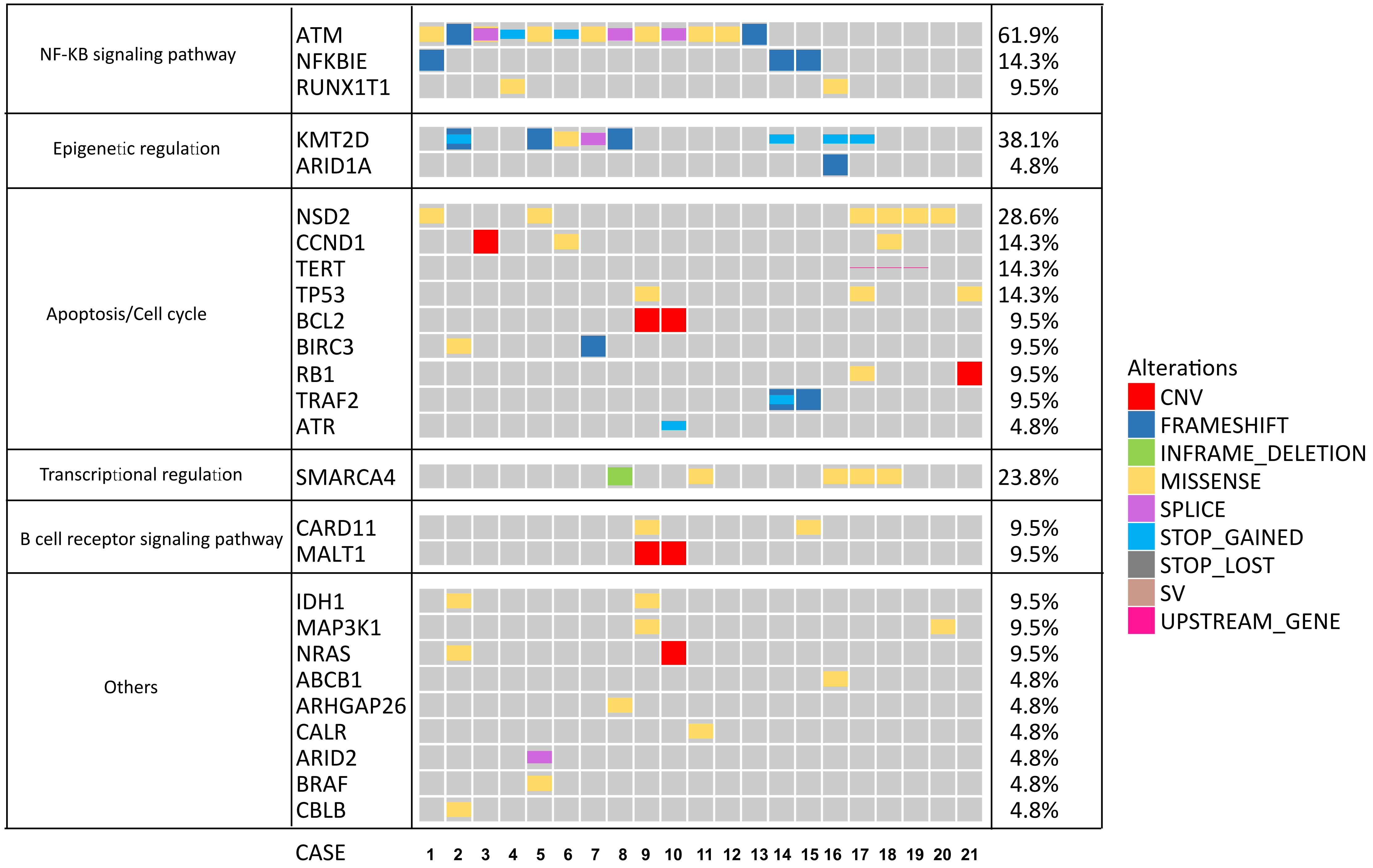
NF-KB signaling pathway
ATM 61.9%
NFKBIE 14.3%
RUNX1T1 9.5%
Epigenetic regulation
KMT2D 38.1%
ARID1A 4.8%
Apoptosis/Cell cycle
NSD2 28.6%
CCND1 14.3%
TERT 14.3%
TP53 14.3%
BCL2 9.5%
BIRC3 9.5%
RB1 9.5%
TRAF2 9.5%
ATR 4.8%
Transcriptional regulation
SMARCA4 23.8%
B cell receptor signaling pathway
CARD11 9.5%
MALT1 9.5%
Others
IDH1 9.5%
MAP3K1 9.5%
NRAS 9.5%
ABCB1 4.8%
ARHGAP26 4.8%
CALR 4.8%
ARID2 4.8%
BRAF 4.8%
CBLB 4.8%
CASE
1 2 3 4 5 6 7 8 9 10 11 12 13 14 15 16 17 18 19 20 21
Alterations
CNV
FRAMESHIFT
INFRAME_DELETION
MISSENSE
SPLICE
STOP_GAINED
STOP_LOST
SV
UPSTREAM_GENE

Figure S3

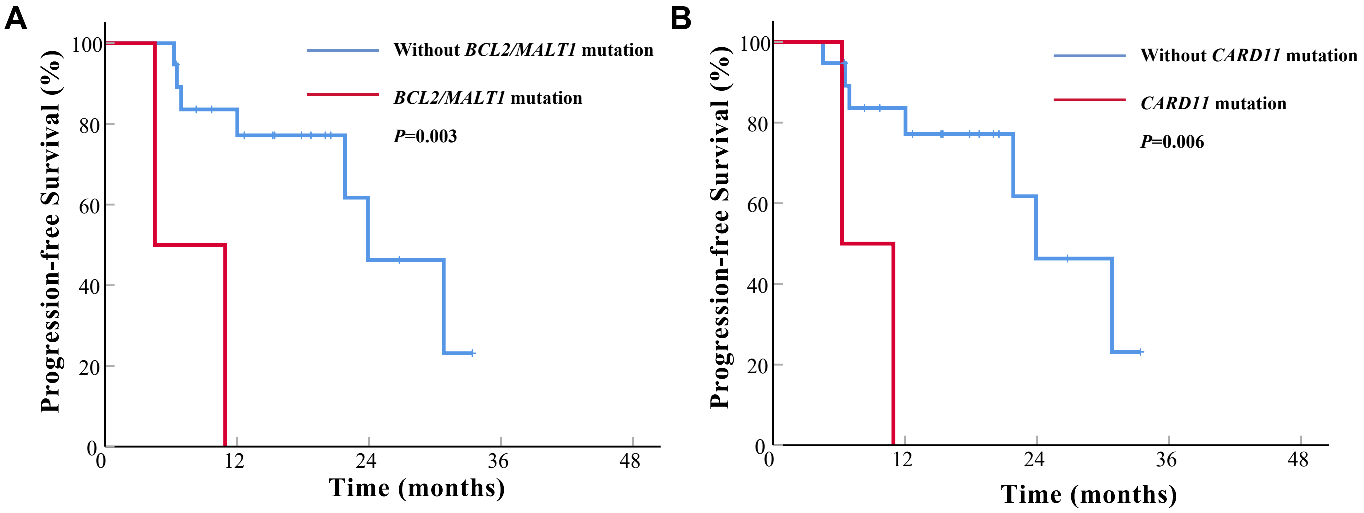

Figure S2. Progression-free survival of patients with or without (A) *BCL2/MALT1* mutation, and (B) *CARD11* mutation. Log-rank *P* values are shown.
